# Patho-mechanisms of the origins of bronchopulmonary dysplasia

**DOI:** 10.1186/s40348-021-00129-5

**Published:** 2021-12-11

**Authors:** Mitali Sahni, Vineet Bhandari

**Affiliations:** 1 Pediatrix Medical Group, Sunrise Children’s Hospital, Las Vegas, NV USA; 2 University of Nevada, Las Vegas, NV USA; 3 Neonatology Research Laboratory, Education and Research Building, Cooper University Hospital, One Cooper Plaza, Camden, NJ 08103 USA

**Keywords:** Preterm lung, Hyperoxia, Placenta, Growth factors, miRNA, Chronic lung disease

## Abstract

Bronchopulmonary dysplasia (BPD) continues to be one of the most common complications of prematurity, despite significant advancement in neonatology over the last couple of decades. The new BPD is characterized histopathologically by impaired lung alveolarization and dysregulated vascularization. With the increased survival of extremely preterm infants, the risk for the development of BPD remains high, emphasizing the continued need to understand the patho-mechanisms that play a role in the development of this disease. This brief review summarizes recent advances in our understanding of the maldevelopment of the premature lung, highlighting recent research in pathways of oxidative stress-related lung injury, the role of placental insufficiency, growth factor signaling, the extracellular matrix, and microRNAs.

## Introduction

Bronchopulmonary dysplasia (BPD) was first described 54 years ago by Northway et al. [1], but it still continues to be one of the most serious causes of mortality and morbidity in the neonatal intensive care unit (NICU). Despite significant advancements in neonatology over the last couple of decades including more gentle methods of invasive mechanical ventilation (IMV), increasing use of non-invasive ventilation, surfactant replacement therapy etc., the incidence of BPD remains quite high [2]. This is partly attributable to the increased survival of extremely low birth weight infants as BPD is more commonly seen in the infants born at < 28 weeks gestational age (GA) [3]. Before the advent of surfactant and gentler ventilation modes, “old BPD” occurred due to oxygen injury and mechanical ventilation and was characterized by abnormal vascularization, with obliteration of vessels and pulmonary fibrosis. On the other hand, the “new BPD” has minimal alveolar septal fibrosis, a lesser degree of airway damage as compared to its old counterpart, and is characterized by alveolar simplification and dysmorphic microvasculature [4]. Other findings reported include bronchial and bronchiolar smooth muscle hyperplasia, as well as altered number of neuroendocrine cells [5]. Although we have made major strides in understanding the patho-mechanics of the origin of BPD, we have limited therapies available to manage established BPD [6, 7]. In addition to the well-known antenatal and perinatal factors that affect BPD, such as genetic susceptibility, immature lung and surfactant homeostasis, intrauterine and perinatal infections [7], recent literature has implicated various mediators of impaired alveolarization and dysregulated vascularization in the perinatal origins of BPD [8–10]. The postnatal therapies used in the NICU to manage respiratory failure with IMV and oxygen use further lead to inflammation and maladapted lung development [6]. This review will focus on studies of lung growth restriction caused by placental insufficiency, the role of placental infection as well as selected mediators of impaired alveolarization and dysregulated vascularization, and their impact on the development of BPD. This narrative review was written after a careful and thorough literature review using PubMed, and focused on published studies in the last 10 years.

### Pathogenesis of BPD

BPD is a complex and multifactorial disease of prematurity, where a multitude of intra-uterine and extra-uterine factors are implicated in its pathogenesis. The strongest predictor of BPD is infant’s GA at birth [11]. The most commonly accepted mechanism of BPD development is ascribed to lung injury and inflammation in immature lungs occurring after post-natal exposure to hyperoxia and IMV, in the presence of placental insufficiency and other predisposing antenatal factors [12]. Recent advances in BPD show that it may occur even after minimal exposure to these post-natal therapies [13]. It is likely that placental insufficiency as well as other placental factors that initiate intrauterine inflammatory processes is interrelated with post-natal exposures in the pathogenesis of BPD [8, 14].

### Role of antenatal placental factors in the development of BPD

#### Inflammation

Increased levels of pro-inflammatory cytokines in amniotic fluid and fetal cord blood are known to be independent risk factors for BPD [13]. A meta-analysis of 59 studies, involving 15,000 infants confirmed an association between histologic chorioamnionitis (CA) and development of BPD [15]. The Alabama Preterm Birth Study, found no association between BPD and CA; however, in the same study, umbilical cord blood culture positive for *Ureaplasma* spp. was associated with an increased risk for BPD [16]. A nested case-control study found a protective effect of CA on BPD in the absence of postnatal sepsis and prolonged ventilation, but demonstrated an increased risk for BPD in infants exposed to histologic CA who were exposed to IMV and postnatal sepsis [17]. Lastly, a cohort study involving preterm infants < 32 weeks gestation, concluded that in infants exposed to histologic CA there was a decreased response to surfactant therapy and prolonged need for ventilation, which led to increased risk for BPD [18]. Hence, we can conclude that CA increases the risk for premature preterm birth, which in turn is one of the most important risk factor for development of BPD. In addition, CA likely induces a chronic inflammatory process that predisposes the lung to post-natal injuries [13]. In preterm infants who later developed BPD, the cord blood levels of inflammatory markers, interleukin-6 (IL-6), and monocyte chemotactic protein-1 (MCP-1) were shown to be significantly elevated [19, 20]. Table 1 lists selected inflammatory mediators in the pathogenesis of BPD and the trends in their expression levels in BPD.

**Table 1:** Selected molecular mediators involved in the pathogenesis of bronchopulmonary dysplasia (BPD). Adapted from [21]

Molecular mediator	Levels associated with increased risk of BPD
*Inflammatory mediators* (*cytokines/chemokines*) [19, 20]	
**IL-6**: interleukin-6	Higher
**MCP-1**: monocyte chemotactic protein-1	Higher
**GCSF**: granulocyte colony stimulating factor	Lower
**IL-1β:** interleukin 1 beta	Higher
**IL-8**: interleukin 8	Higher
**TNF-α**: tumor necrosis factor alpha	Higher
**IFNγ:** interferon gamma	Higher
*Growth factors*	
**TGF β**: transforming growth factor β [22]	Higher
**CTGF**: connective tissue growth factor [23]	Higher
**PDGF**: platelet-derived growth factor BB [24]	Higher
**KGF**: keratinocyte growth factor [25]	Lower
**HGF:** hepatocyte growth factor [26]	Lower
**FGF:** fibroblast growth factor [27]	Lower
*Vascular mediators*	
**VEGF**: Vascular endothelial growth factor [28]	Variable
**Ang 2**: angiopoetin 2 [29]	Higher

#### Role of chronic placental insufficiency

Several epidemiologic studies have demonstrated associations between intrauterine growth restriction (IUGR), preeclampsia (PE), hypertensive disorders of pregnancy, maternal smoking, and other antenatal factors with susceptibility to BPD [30]. It is postulated that if there is sustained disruption of lung development initially, it can lead to abnormal lung structure, even in the absence of additional postnatal stressors, e.g., exposure to hyperoxia and IMV. Alternatively, interactions between antenatal stresses may alter susceptibility to postnatal stressors, thereby increasing the risk of developing BPD [31]. An observational study reported a twofold increase in mortality (both early and late) and increased risk for BPD in small for gestational age (SGA) infants born at or below 32 weeks GA [32]. Another study demonstrated that preterm infants born with IUGR, remain at high risk for late respiratory morbidities and lung function at school age [33]. The ELGAN investigators evaluated prenatal predictors of BPD in neonates born before 28 weeks GA and found fetal growth restriction to be an independent risk factor for BPD, after controlling for other risk factors. In this study, markers of placental inflammation and infection were not associated with increased risk of BPD [34]. In a separate cohort of neonates born at GA < 29 weeks, Keller et al. showed that IUGR was associated with increased odds of persistent respiratory morbidity at 1-year corrected age [35].

These studies implicate the critical role of altered placental structure and function in the pathobiology of BPD. Placental insufficiency related to IUGR and PE is associated with fetal growth restriction. Experimental studies have demonstrated impaired angiogenic and growth properties of endothelial cells derived from human IUGR placentas, which may be related to decreased aryl hydrocarbon receptor nuclear translocator (ARNT) expression [36]. Further work on cord blood biomarkers showed that decreased vascular endothelial growth factor (VEGF) and soluble VEGF receptor 1 (sFlt-1) levels are strongly associated with IUGR and are predictive of BPD in preterm infants [37]. Mestan et al. demonstrated that examination of placental tissue for vascular lesions that reflect maternal vascular underperfusion (MVU) after preterm birth provides a unique approach to predict the subsequent risk for BPD [38]. They also reported that cord blood biomarkers, like granulocyte colony stimulating factor, placental growth factor, and VEGF-A are decreased in association with placental findings of MVU and are associated with subsequent diagnoses of BPD [39]. The developing fetal lung may share certain structural and functional aspects with the placenta. These anatomical and pathophysiological similarities between placenta and lung may reflect changes in the developing premature lung, and may present an important insight in understanding the neonatal lung biology [31]. Various cord blood biomarkers studied in preterm infants, such as decreased VEGF, that is suggestive of impaired angiogenesis, is also strongly linked with findings of placental vascular lesions on histology and is further associated with a higher risk for BPD with pulmonary hypertension (PH) [37, 39, 40]. Table 1 lists selected molecular mediators in the pathogenesis of BPD and the trends in their expression levels in BPD.

### Mediators of impaired alveolarization and dysregulated vascularization in BPD

#### Oxygen toxicity

**Fig. 1:**
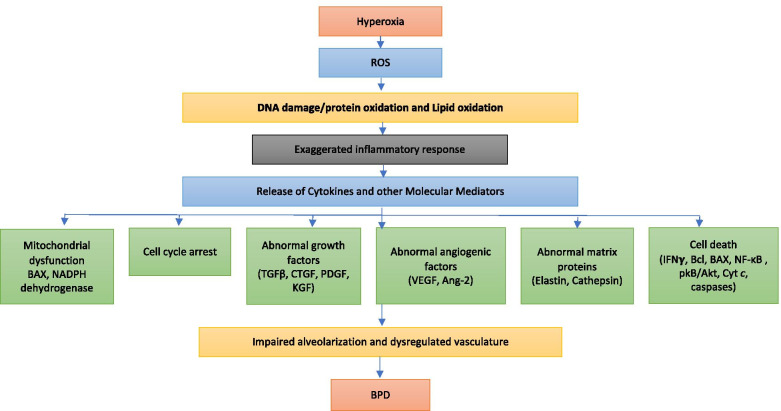
Exposure to hyperoxia leads to production of reactive oxygen species (ROS) that leads to an exaggerated inflammatory response, and releases cytokines and other molecular mediators. Subsequently, this causes abnormal responses of angiogenic factors, growth factor signaling, abnormal matrix protein formation, mitochondrial dysfunction, cell cycle arrest, and cell death. This, in turn, causes impaired alveolarization and dysregulated vasculature which is pathognomic of bronchopulmonary dysplasia (BPD). BAX, bcl-2 like protein; NADPH: nicotinamide adenine dinucleotide phosphate; TGFβ, transforming growth factor β; CTGF, connective tissue growth factor; PDGF, platelet-derived growth factor; KGF, keratinocyte growth factor; VEGF, vascular endothelial growth factor; Ang-2, angiopoietin 2; Cyt *c*, cytochrome *c*; IFNγ, interferon gamma; NF-κB, nuclear factor-kappa B; pkB, protein kinase B

Oxygen therapy is one of the mainstays of management of acute respiratory failure in preterm neonates. However, it comes at the expense of oxidative stress to the immature lungs. Acute lung injury secondary to hyperoxia is characterized by an inflammatory response with disruption of the alveolar-capillary barrier, influx of inflammatory mediators, vascular leak, and pulmonary edema, ultimately leading to cell death [41, 42]. In a neonatal mouse model of BPD, hyperoxia exposure in the critical saccular stage of lung development reproduces the changes seen in human BPD and these effects were found to be dose-dependent on the fraction of inspired oxygen being administered [9]. Figure 1 provides an overview of the mechanisms involved in hyperoxia-induced lung cell injury and cell death.

Based on animal studies, it is postulated that hyperoxia exposure to preterm lung leads to release of vascular mediators like angiopoietin 2 (Ang2) and VEGF that disrupts the alveolar-capillary membrane leading to pulmonary edema and contributes to lung injury [29, 43]. Ang2 is an angiogenic growth factor that is known to destabilize blood vessels, increase vascular leak and enhance vascular regression and endothelial cell apoptosis. VEGF is expressed in lungs and promotes endothelial cell growth and remodeling [43]. Hyperoxia exposure can also release other pro-inflammatory cytokines such as IL-1β [44], IL-6 [45], interferon gamma (IFN-γ) [46], tumor necrosis factor alpha (TNF-α) [47], and IL-8 [43, 48] that can invoke significant damage to the capillary and the alveolar epitheliums, worsening lung injury [49, 50]. The abnormal lung architecture with increased cell death mediated by IFN-γ has been shown to be dependent on matrix metalloproteinase 9 (MMP9) in murine lungs exposed to hyperoxia [51]. Table 1 lists selected molecular mediators in the pathogenesis of BPD and the trends in their expression levels in BPD. Inflammatory cells as well as hyperoxia per se release reactive oxygen species (ROS). Studies have shown a reduced level of antioxidants in preterm infant that develop BPD [52]. When the production of ROS exceeds the antioxidant capacity of the cell, it results in oxidative stress, which then leads to cellular and tissue injury via lipid peroxidation, DNA damage, and protein oxidation [53]. These ROS further cause cell death by activating key caspases and triggering surface death receptors like Fas in the extrinsic pathway or via the mitochondrial cell death pathway in which Bax proteins interact with or form mitochondrial pores, release cytochrome *c*, activate caspase-9, and induce cell death [9]. Based on studies in mice models of BPD, and on cultures of pulmonary endothelial cells, we also know that hyperoxia primarily affects complex 1 (NADH dehydrogenase) activity, causing inhibition of NADH-linked mitochondrial respiration [54]. Even partial pharmacological inhibition of complex 1 by pyridaben (a selective inhibitor of complex 1) in neonatal mice can produce the phenotype of arrested alveolar development that is seen with hyperoxia exposure [54, 55]. In another study in rodents, inhibition of mitochondrial respiratory complex III by antimycin A led to chronic mitochondrial dysfunction that induced vascular damage and metabolic reprogramming that may have induced pulmonary hypertension [56].

Cellular injury in BPD can also be amplified by gap-junction (GJ) mediated intercellular communication [57]. Oxidative stress can cause increase in Connexin 43 (Cx43) expression, which is a critical GJ protein. In rat model of BPD, Jianhua et al. showed that increased Cx43-GJ-mediated intercellular communication induces excessive apoptosis via the ASK1-JNK/p38 signaling pathway. They further showed that use of Gap26, a Cx43-GJ inhibitor can reverse these changes and improve alveolar development in neonatal rats exposed to hyperoxia [58]. In pre-clinical models of BPD in guinea pigs, use of oxidized glutathione as a supplement led to improved alveolar development [59]. In a recent study by Shrestha et al., a combined transcriptomic and proteomic analysis was done to evaluate the effect of hyperoxia exposure during the saccular and alveolar stages of lung development, on the expression level of genes and proteins necessary for optimal lung development and repair. The authors report that hyperoxia exposure dysregulated the expression of 344 genes and 21 proteins, which were involved in maturation of lung tissue. Oxidoreductase activity, plasma membrane integrity, apoptosis, organ development, cell proliferation, angiogenesis, and mitophagy were found to be affected by hyperoxia [60]. Other key regulators of cell death include members of the Bcl-2 gene family (mainly anti-apoptotic Bcl-2 and Bcl-x_L_, pro-apoptotic Bax-type proteins, and pro-apoptotic BH3-domain-only members), transcription factor, nuclear factor kappa B (NF-κB), and protein kinase B/Akt [9].

Genetic studies have also documented antioxidant response genes like the rs6721961 single nucleotide polymorphism (SNP) in the *NFE2L2* gene, which was associated with decreased incidence of severe BPD in human infants [61]. Another study explored the role of superoxide dismutases (SODs), in which loss of SOD3 (in *Sod3* ^−/−^ mice) worsened alveolarization, which was intensified after bleomycin application [62]. In summary, hyperoxia-induced injury along with a simultaneous attempt at repair results in lung pathology with the characteristic features of BPD in preterm infants.

#### Growth factor signaling in BPD

Growth factors are considered important mediators of dysregulated lung development seen in BPD. One of the most studied growth factors is the transforming growth factor (TGF)-β [63]. Lung injury and inflammation leads to induction of TGF-β that curbs the inflammatory processes, and plays a pivotal role in mediating lung repair and tissue remodeling [64]. As opposed to fibroblast growth factor (FGF)-β and IL-1β, both of which lower elastin mRNA levels in human lung fibroblasts, TGF-β has been shown to increase elastin by transcriptional and posttranscriptional mechanisms [65]. Increased TGF-β signaling has been implicated in resulting in the BPD phenotype [22], with neutralizing antibodies against it shown to improve hyperoxia-induced lung injury [66]. Recent studies have also suggested a role for the TGF-β-induced protein in impaired lung alveolarization [67]. Deng et al. also reported excessive activation of TGF-β signaling caused impaired alveolar development, and elastin deposition in the newborn mouse lung exposed to hyperoxia. In their study, use of TGF-β neutralizing antibodies-1D11 led to improved alveolarization, as well as the distribution of elastin in hyperoxia-exposed newborn lung. The expression levels of tropoelastin, fibulin-5, and neutrophil elastase, which are important components of elastogenesis, were decreased by treatment with 1D11 in the injured newborn lung [68].

Studies on the TGF-β system have also included TGF-β ligand/receptors, as well as latent TGF-β-binding protein 4, which regulates “inactive” TGF-β that is embedded in the extracellular matrix [10]. Recent work on stem-cell therapy has also highlighted the pathogenic role of TGF-β signaling, where application of bone marrow mesenchymal stem cells (via the intravenous route), with concomitant erythropoietin administration (via the intraperitoneal route) led to improved alveolarization in a hyperoxia-based mouse model of BPD. Improved alveolarization was also accompanied by reduced TGF-β1 levels, and reduced proximal TGF-β signaling [69]. Although most studies have only focused on the excessive TGF-β activation in the pathogenesis of BPD, Conway et al. proposed that neonatal hyperoxia exposure initially diminishes saccular TGF-β signaling that coincides with alveolar simplification. In their mouse model of BPD, they have shown that at multiple levels during the period of postnatal lung development, TGF-β ligands, intracellular mediators, and downstream TGF-β–responsive extracellular matrix targets, are all suppressed initially and contribute to the arrested alveolar septation [67].

Altered expression of TGF-β [70], as well as lower levels of various pulmonary and vascular growth factors, continues to be implicated in the pathogenesis of BPD [71]. Similar to TGF-β, overexpression of platelet-derived growth factor-BB has also been shown to result in pulmonary fibrosis [24]. Keratinocyte and hepatocyte growth factors are also thought to participate in normal lung development and tissue regeneration after lung injury. Low airway concentrations of keratinocyte and hepatocyte growth factors have been found to be associated with BPD and may play a role in the pathogenesis of BPD [25, 26]. Based on a study in an experimental mouse model of saccular lung development, FGF-10 signaling was shown to be crucial for development of saccular airway [27]. FGF-10 expression was decreased in human lung tissue samples of infants with BPD. Furthermore, inhibition of FGF-10 by inflammatory signaling involves the NF-κB-dependent interactions between RELA, SP3, and the FGF-10 promoter [72].

Among the various angiogenic growth factors, VEGF has received significant importance due to its critical role in vascular development and presence in highly vascularized tissues [9, 13, 73]. It has been identified as a key regulator of angiogenesis and lung maturation due to its role in coordinating airway branching and angiogenesis [74]. Disturbance in VEGF signaling can lead to impaired lung parenchymal development and long-term lung injury [75]. VEGF levels were increased in newborn rabbits after hyperoxia exposure; however, in a similar setting, they were shown to be decreased in the premature fetal baboon model [43]. When comparing studies, it is important to be aware of the temporal relationship between VEGF levels and stages of lung development/injury in animal models as well as humans developing BPD [28]. VEGF levels were reported to be significantly lower in preterm infants who developed BPD, when compared to infants who recovered from ventilator and hyperoxia-induced lung injury [76]. Inhibition of VEGF receptor led to impaired alveolarization and lung vascular growth with PH [77]. In 2 studies in mouse models of BPD, VEGF gene therapy has been shown to improve survival, promoted lung capillary formation, and improved alveolarization [75, 78]. It also increased alveolar nitric oxide synthase (NOS) expression, which indicates that the favorable effect of VEGF may be NO mediated. VEGF overexpression in newborn mice induces inducible NOS (iNOS) and eNOS-dependent lung simplification, pulmonary edema, and oxidant stress. In VEGF transgenic mice, NOS inhibition can decrease oxidative stress, vascular permeability, and angiogenesis [79]. Pathological examination of lungs in infants that died of BPD, showed defective alveolar septation and capillary formation, as well as reduced expression of VEGF and VEGF receptor 1 (VEGF-R1). Altered VEGF signaling and TGFβ activation reduces the expression of VEGF-R2 in endothelial cells, contributing to the defective lung morphology seen after prolonged mechanical ventilation [80].

Another key mediator of pulmonary fibrosis, as seen in a sheep model of BPD, is connective tissue growth factor (CTGF), which is responsible for the downstream effects of TGFβ [81].

In addition to producing fibrosis, CTGF can also prolong wound healing. TGF-β1 induces CTGF in endothelial cells and fibroblasts. In sheep, endotoxin-induced inflammation leads to increased TGF-β1 expression and reduction in CTGF, which may affect vascular development [82]. CTGF, also known as CCN2, is required for normal lung development. Lung tissue from infants with BPD demonstrates increased expression of CTGF [83, 84]. Increased CTGF expression induced by hyperoxia, inflammation, and mechanical ventilation can disrupt alveologenesis and capillary formation, and induce fibrosis during the critical period of alveolar development [23]. Overexpression of CTGF in alveolar epithelial type II cells disrupts alveolarization and vascular development, and can result in pulmonary vascular remodeling and PH [85]. In a rodent model of hyperoxia-induced BPD, inhibition of CTGF by a CTGF monoclonal antibody prevented β-catenin signal activation, improved alveolarization and vascular development, and decreased pulmonary vascular remodeling and PH [84]. Table 1 lists selected growth factors in the pathogenesis of BPD and the trends in their expression levels in BPD.

#### microRNAs

Recently microRNAs (miRs) have been described as important moderators of normal growth, development, and disease [86]. In a neonatal mouse hyperoxia model of BPD, Syed et al. demonstrated significantly increased levels of miR-34a levels; further, deletion or inhibition of miR-34a improved the pulmonary phenotype and BPD-associated pulmonary arterial hypertension (PAH) in the mouse models of BPD [87]. Epithelial miR-34a was shown as a pathogenic mediator of abnormal alveolarization, with Ang1 as the relevant miR-34a target. Additionally, the utility of an antagomir targeting miR-34a was a candidate intervention, to promote proper lung alveolarization. The role of miR-34a in the pathogenesis of BPD has been independently confirmed, with the focus on targeting platelet-derived growth factor receptor (PDGFR)α, in an experimental model of BPD [88]. This was studied using target site blockers, which are antisense oligonucleotides that bind to miR target site of a mRNA, preventing miR from gaining access to that site. This allows us to study the effects of the miR on a single target. Hence, pharmacologic inhibition of miR-34a can be used as a therapeutic option in neonates to prevent hyperoxia- induced lung injury [89, 90]. In another study, increased expression of miR-451 was noted in a mouse model of BPD and inhibition of miR-451 was shown to improve the cardiopulmonary phenotype [91].

Similarly, potential roles of miR-17∼92 [92], miR-29b [93], miR-876-3p [94], miR-199a-5p [95], and miR-489 [96] have been described in the pathogenesis of BPD. Decreased plasma levels of miR-17 were seen in clinical subjects who developed BPD and the levels correlated with disease severity [97]. In a hyperoxia-based experimental mouse model of BPD where systemic inflammation was induced, a similar decrease in levels of miR-17-92 cluster was noted [92].

Plasma levels of miR-29b are suppressed in the first week of life in preterm infants that subsequently develop BPD and the decrease inversely correlated with BPD severity. Similarly decreased pulmonary miR-29b expression is seen in a mouse model of BPD [98].

Furthermore, adeno-associated 9 (AAV9)-mediated restoration of miR-29b in the developing lung in a mouse model led to modest improvements in alveolarization and completely attenuated the defects in matrix protein expression and localization [93]. Reduced miR-876-3p is seen in BPD-susceptible, compared with BPD-resistant infants, and was confirmed in the animal model/in vitro models of BPD. In the in vivo BPD model, gain of function of miR-876-3p improved the alveolar architecture, demonstrating a causal link between miR-876-3p and BPD [94]. Enhanced expression of miR-199a-5p has been reported in hyperoxia-exposed mice lungs and cells, and in tracheal aspirates of infants developing BPD. miR-199a-5p-mimic worsens hyperoxia-induced acute lung injury (HALI) and miR-199a-5p-inhibitor treatment attenuated HALI [95]. Lower levels of miR-489 have been noted in the lungs of infants with BPD and in a hyperoxia-induced mouse model of BPD, and it has been demonstrated as a possible inhibitor of alveolar septation [96]. In another study, miR-154 overexpression was associated with enhanced TGF-β signaling in the lung and led to a BPD-like phenotype characterized by alveolar simplification [99].

#### The extracellular matrix

Various studies have explored the role of extracellular matrix (ECM) in association with abnormal lung alveolarization seen with BPD [100]. Continued dynamic changes in the expression of the molecules in ECM, e.g., collagen, elastin, enzymes such as metalloproteinases [101] and neutrophil elastase [102], and ECM cross linking enzymes play a pivotal role in lung development [103]. Luan et al. have described the spatial and temporal changes in extracellular elastin and laminin distribution during lung alveolar development [104]. Essentially, in aberrant lung development, the overall amount of elastin is reduced, elastin fibers in the septa are disorganized [105]; although the amount of collagen and collagen crosslinks is increased, the collagen fibers have abnormal structure [106].

However, problems with elastin formation alone have not been shown to cause abnormal alveolarization. So, studies have targeted ECM crosslinking machinery, including lysyl oxidases and transglutaminases [107]. Lysyl oxidase expression in lungs was deregulated in mice after hyperoxia, but pharmacological inhibition of lysyl oxidase did not improve the aberrant lung architecture, although the collagen levels were partially restored, and the distribution and morphology of lung elastin fibers were improved [105]. Transglutaminases 2 (TGM2) was increased in expression in an experimental animal model of BPD and targeted deletion of TGM2 in C57BL/6J mice increased septal thickness and reduced gas-exchange surface area. During hyperoxia, collagen structures in TGM2^−/−^ mice were partially protected from hyperoxia effects, i.e., collagen crosslinking partially improved; however, the lung alveolar architecture was not restored. Pharmacological inhibition of TGM restored normal collagen crosslink under pathological conditions and also led to moderate improvement in alveoli size and gas-exchange surface density [108]. It appears that interfering in a single ECM-crosslinking system may not be sufficient to show sustained improvement in dysregulated alveolarization but a multi-pronged approach may help [10].

### Novel techniques and resources influencing our understanding of the pathogenesis of BPD

The lung is a complex structure, consisting of a wide variety of cells and processes that play a vital role in lung maturation and adaptation. However, most genomic and proteomic studies use bulk measurements from whole lung tissue to evaluate perinatal lung development, thus limiting insight into the interactions and purpose of individual cells [109]. Single cell RNA-seq (scRNA-seq) allows transcriptomic mapping of individual cells to understand cellular heterogeneity and responses in complex biological systems [110]. Using scRNA profiling, we can identify varied pulmonary cell lines during perinatal development, and provide information on genes, processes, and cell–cell interactions that regulate pulmonary structure and function at birth [111]. In a recent study, Guo et al. utilized scRNA-seq on post-natal day 1 mouse lung with developmental RNA profiles obtained from whole lung tissue, to understand the complexity of cellular adaptation of the lung to air breathing at the time of birth. To facilitate the inquiry and re-utilization of their data, the authors also developed a web application, named “single cells of Lung At Birth” (scLAB), that allows query by gene of interest, cell type, or dynamic gene expression patterns during mouse lung development and is freely accessible [111].

Another great resource for investigators is LungMAP (https://www.lungmap.net/) that includes a variety of a searchable databases that address transcriptomics, proteomics, and lipidomics, as well as includes an image database [112]. As part of the LungMAP consortium, a facility has now been developed that also provides tissue and cells from developing human lungs to investigators, through the Biorepository for Investigation of Neonatal Diseases of the Lung [10]. In a recent study, researchers utilized the interactive Dynamic Regulatory Events Miner (iDREM) method to reconstruct a dynamic model, that identifies the grouping of genes during the development of lung into transcription factors, miRs, and methylation events that regulate them. They also developed an interactive tool that allows users to query, and obtain information on specific genes, miRs, regulators, etc., and enables them to integrate other types of data, like single-cell and sorted-cell RNA-Seq data with the model. This interactive model revealed both genes and functions involved in alveolar development and identified new roles for several transcription factors and miRs in regulating various stages of this process [113].

## Conclusions

The pathogenesis of BPD involves a multifactorial pathway where lung injury occurs over time with hyperoxia and IMV in the setting of preexisting prenatal determinants, leading to dysregulated immune responses and aberrant tissue repair in preterm infants. This leads to impaired alveolarization and vascular development. Continued research has provided insight into various pathways involved in pathogenesis of BPD. Recent literature has focused on the role of oxidative stress-related genes, growth factor signaling, miRs, ECM modeling, and modulation of inflammation. A more robust understanding of the mechanistic contribution of miRs in regulating aberrant alveolarization and vascular development in BPD has provided means for a tantalizing new therapeutic pathway in BPD research. We hope future research in investigating the molecular mechanisms in BPD will ultimately help develop targeted therapeutic strategies for this devastating disease.

## Data Availability

Not applicable.

## Abbreviations

AAV: Adeno-associated virus
Ang2: Angiopoietin 2
ARNT: Aryl hydrocarbon receptor nuclear translocator
Bax: Bcl-2 associated X (protein)
BPD: Bronchopulmonary dysplasia
CA: Chorioamnionitis
CTGF: Connective tissue growth factor
Cx43: Connexin 43
DNA: Deoxyribonucleic acid
ECM: Extracellular matrix
ELGAN: Extremely low gestational age newborns
GA: Gestational age
GJ: Gap junction
HALI: Hyperoxia-induced acute lung injury
IL: Interleukin
IFN-γ: Interferon-gamma
IMV: Intermittent mandatory ventilation
IUGR: Intrauterine growth restriction
MCP-1: Monocyte chemotactic protein-1
miR: Micro ribonucleic acid
MMP: Matrix metalloproteinase
MVU: Maternal vascular underperfusion
NADH: Nicotinamide adenine dinucleotide + hydrogen
NF-κB: Nuclear factor kappa B
NICU: Neonatal intensive care unit
NOS: Nitric oxide synthase
PE: Preeclampsia
PAH: Pulmonary arterial hypertension
PDGFR: Platelet-derived growth factor receptor
PH: Pulmonary hypertension
ROS: Reactive oxygen species
scRNA: Single cell ribonucleic acid
SGA: Small for gestational age
SNP: Single nucleotide polymorphism
SOD: Superoxide dismutase
TGF-β: Transforming growth factor-beta
TNF-α: Tumor necrosis factor-alpha
TGM: Transglutaminase
VEGF: Vascular endothelial growth factor
VEGFR1: Vascular endothelial growth factor receptor 1; also known as “soluble FMS-like tyrosine-kinase1” or sFLT1

## References

[1] Northway WH, Rosan RC, Porter DY (1967). Pulmonary disease following respirator therapy of hyaline-membrane disease. Bronchopulmonary dysplasia. N Engl J Med.

[2] Hwang JS, Rehan VK (2018). Recent advances in bronchopulmonary dysplasia: pathophysiology, prevention, and treatment. Lung..

[3] Stoll BJ, Hansen NI, Bell EF (2015). Trends in care practices, morbidity, and mortality of extremely preterm neonates, 1993-2012. Jama..

[4] Husain AN, Siddiqui NH, Stocker JT (1998). Pathology of arrested acinar development in postsurfactant bronchopulmonary dysplasia. Hum Pathol.

[5] De Paepe ME, Bhandari V (2016). Pathology of bronchopulmonary dysplasia. Bronchopulmonary Dysplasia.

[6] Bhandari A, Bhandari V (2009). Pitfalls, problems, and progress in bronchopulmonary dysplasia. Pediatrics..

[7] Sahni M, Bhandari V (2020) Recent advances in understanding and management of bronchopulmonary dysplasia. F1000Res 9:F1000 Faculty Rev-703 10.12688/f1000research.25338.1 PMC7361502 32704351

[8] Mestan KK, Steinhorn RH (2011). Fetal origins of neonatal lung disease: understanding the pathogenesis of bronchopulmonary dysplasia. Am J Physiol Lung Cell Mol Physiol.

[9] Bhandari V (2010). Hyperoxia-derived lung damage in preterm infants. Semin Fetal Neonatal Med.

[10] Morty RE (2018). Recent advances in the pathogenesis of BPD. Semin Perinatol.

[11] Jensen EA, Schmidt B (2014). Epidemiology of bronchopulmonary dysplasia. Birth Defects Res A Clin Mol Teratol.

[12] Sahni M, Mowes AK (2019). Bronchopulmonary dysplasia. StatPearls.

[13] Glaser K, Speer CP, Bhandari V (2016). Pre and postnatal inflammation in the pathogenesis of bronchopulmonary dysplasia. Bronchopulmonary Dysplasia.

[14] Balany J, Bhandari V (2015). Understanding the impact of infection, inflammation, and their persistence in the pathogenesis of bronchopulmonary dysplasia. Front Med.

[15] Hartling L, Liang Y, Lacaze-Masmonteil T (2012). Chorioamnionitis as a risk factor for bronchopulmonary dysplasia: a systematic review and meta-analysis. Arch Dis Child Fetal Neonatal Ed.

[16] Goldenberg RL, Andrews WW, Goepfert AR (2008). The Alabama Preterm Birth Study: umbilical cord blood Ureaplasma urealyticum and Mycoplasma hominis cultures in very preterm newborn infants. Am J Obstet Gynecol.

[17] Van Marter LJ, Dammann O, Allred EN (2002). Chorioamnionitis, mechanical ventilation, and postnatal sepsis as modulators of chronic lung disease in preterm infants. J Pediatr.

[18] Been JV, Rours IG, Kornelisse RF, Jonkers F, de Krijger RR, Zimmermann LJ (2010). Chorioamnionitis alters the response to surfactant in preterm infants. J Pediatr.

[19] Matoba N, Yu Y, Mestan K (2009). Differential patterns of 27 cord blood immune biomarkers across gestational age. Pediatrics..

[20] Otsubo Y, Hashimoto K, Kanbe T, Sumi M, Moriuchi H (2017). Association of cord blood chemokines and other biomarkers with neonatal complications following intrauterine inflammation. PLoS One.

[21] Bhandari A, Bhandari V (2013). Biomarkers in bronchopulmonary dysplasia. Paediatr Respir Rev.

[22] Alejandre-Alcázar MA, Kwapiszewska G, Reiss I (2007). Hyperoxia modulates TGF-beta/BMP signaling in a mouse model of bronchopulmonary dysplasia. Am J Physiol Lung Cell Mol Physiol.

[23] Wu S, Platteau A, Chen S, McNamara G, Whitsett J, Bancalari E (2010). Conditional overexpression of connective tissue growth factor disrupts postnatal lung development. Am J Respir Cell Mol Biol.

[24] Adcock KG, Martin J, Loggins J, Kruger TE, Baier RJ (2004). Elevated platelet-derived growth factor-BB concentrations in premature neonates who develop chronic lung disease. BMC Pediatr.

[25] Danan C, Franco M-L, Jarreau P-H (2002). High concentrations of keratinocyte growth factor in airways of premature infants predicted absence of bronchopulmonary dysplasia. Am J Respir Crit Care Med.

[26] Lassus P, Heikkilä P, Andersson LC, von Boguslawski K, Andersson S (2003). Lower concentration of pulmonary hepatocyte growth factor is associated with more severe lung disease in preterm infants. J Pediatr.

[27] Benjamin JT, Smith RJ, Halloran BA, Day TJ, Kelly DR, Prince LS (2007). FGF-10 is decreased in bronchopulmonary dysplasia and suppressed by Toll-like receptor activation. Am J Physiol-Lung CelL Mol Physiol.

[28] Meller S, Bhandari V (2012). VEGF levels in humans and animal models with RDS and BPD: temporal relationships. Exp Lung Res.

[29] Bhandari V, Choo-Wing R, Lee CG (2006). Hyperoxia causes angiopoietin 2-mediated acute lung injury and necrotic cell death. Nat Med.

[30] Manuck TA, Levy PT, Gyamfi-Bannerman C, Jobe AH, Blaisdell CJ (2016). Prenatal and perinatal determinants of lung health and disease in early life: a National Heart, Lung, and Blood Institute workshop report. JAMA Pediatr.

[31] Taglauer E, Abman SH, Keller RL (2018). Recent advances in antenatal factors predisposing to bronchopulmonary dysplasia. Semin Perinatol.

[32] Lal MK, Manktelow BN, Draper ES, Field DJ (2003). Chronic lung disease of prematurity and intrauterine growth retardation: a population-based study. Pediatrics..

[33] Ronkainen E, Dunder T, Kaukola T, Marttila R, Hallman M (2016). Intrauterine growth restriction predicts lower lung function at school age in children born very preterm. Arch Dis Child Fetal Neonatal Ed.

[34] Bose C, Van Marter LJ, Laughon M (2009). Fetal growth restriction and chronic lung disease among infants born before the 28th week of gestation. Pediatrics..

[35] Keller RL, Feng R, DeMauro SB (2017). Bronchopulmonary dysplasia and perinatal characteristics predict 1-year respiratory outcomes in newborns born at extremely low gestational age: a prospective cohort study. J Pediatr.

[36] Su EJ, Xin H, Yin P (2015). Impaired fetoplacental angiogenesis in growth-restricted fetuses with abnormal umbilical artery doppler velocimetry is mediated by aryl hydrocarbon receptor nuclear translocator (ARNT). J Clin Endocrinol Metab.

[37] Voller SB, Chock S, Ernst LM (2014). Cord blood biomarkers of vascular endothelial growth (VEGF and sFlt-1) and postnatal growth: a preterm birth cohort study. Early Hum Dev.

[38] Mestan KK, Check J, Minturn L (2014). Placental pathologic changes of maternal vascular underperfusion in bronchopulmonary dysplasia and pulmonary hypertension. Placenta..

[39] Mestan KK, Gotteiner N, Porta N, Grobman W, Su EJ, Ernst LM (2017). Cord blood biomarkers of placental maternal vascular underperfusion predict bronchopulmonary dysplasia-associated pulmonary hypertension. J Pediatr.

[40] Check J, Gotteiner N, Liu X (2013). Fetal growth restriction and pulmonary hypertension in premature infants with bronchopulmonary dysplasia. J Perinatol.

[41] Warner BB, Stuart LA, Papes RA, Wispe JR (1998). Functional and pathological effects of prolonged hyperoxia in neonatal mice. Am J Physiol.

[42] Harijith AK, Bhandari V, Bhandari V (2016). Hyperoxia in the pathogenesis of bronchopulmonary dysplasia. Bronchopulmonary Dysplasia.

[43] Bhandari V, Elias JA (2006). Cytokines in tolerance to hyperoxia-induced injury in the developing and adult lung. Free Radic Biol Med.

[44] Bry K, Whitsett JA, Lappalainen U (2007). IL-1beta disrupts postnatal lung morphogenesis in the mouse. Am J Respir Cell Mol Biol.

[45] Choo-Wing R, Nedrelow JH, Homer RJ, Elias JA, Bhandari V (2007). Developmental differences in the responses of IL-6 and IL-13 transgenic mice exposed to hyperoxia. Am J Physiol-Lung CelL Mol Physiol.

[46] Choo-Wing R, Syed MA, Harijith A (2013). Hyperoxia and interferon-γ-induced injury in developing lungs occur via cyclooxygenase-2 and the endoplasmic reticulum stress-dependent pathway. Am J Respir Cell Mol Biol.

[47] Allen GL, Menendez IY, Ryan MA (2000). Hyperoxia synergistically increases TNF-alpha-induced interleukin-8 gene expression in A549 cells. Am J Physiol Lung Cell Mol Physiol.

[48] Thompson A, Bhandari V (2008). Pulmonary biomarkers of bronchopulmonary dysplasia. Biomark Iinsights.

[49] Iliodromiti Z, Zygouris D, Sifakis S (2013). Acute lung injury in preterm fetuses and neonates: mechanisms and molecular pathways. J Matern Fetal Neonatal Med.

[50] Sahni M, Yeboah B, Das P et al (2020) Novel biomarkers of bronchopulmonary dysplasia and bronchopulmonary dysplasia-associated pulmonary hypertension. J Perinatol 40(11):1634–1643 10.1038/s41372-020-00788-8 PMC7664991 32811975

[51] Harijith A, Choo-Wing R, Cataltepe S (2011). A role for matrix metalloproteinase 9 in IFNγ-mediated injury in developing lungs: relevance to bronchopulmonary dysplasia. Am J Respir Cell Mol Biol.

[52] Abdel Ghany EA, Alsharany W, Ali AA, Youness ER, Hussein JS (2016). Anti-oxidant profiles and markers of oxidative stress in preterm neonates. Paediatr Int Child Health.

[53] Buczynski BW, Maduekwe ET, O’Reilly MA (2013). The role of hyperoxia in the pathogenesis of experimental BPD. Semin Perinatol.

[54] Ratner V, Starkov A, Matsiukevich D, Polin RA, Ten VS (2009). Mitochondrial dysfunction contributes to alveolar developmental arrest in hyperoxia-exposed mice. Am J Respir Cell Mol Biol.

[55] Ten VS, Ratner V (2020). Mitochondrial bioenergetics and pulmonary dysfunction: current progress and future directions. Paediatr Respir Rev.

[56] Rafikova O, Srivastava A, Desai AA, Rafikov R, Tofovic SP (2018). Recurrent inhibition of mitochondrial complex III induces chronic pulmonary vasoconstriction and glycolytic switch in the rat lung. Respir Res.

[57] Spray DC, Hanstein R, Lopez-Quintero SV, Stout RF, Suadicani SO, Thi MM (2013). Gap junctions and bystander effects: good Samaritans and executioners. Wiley Interdiscip Rev Membr Transp Signal.

[58] Qing C, Xinyi Z, Xuefei Y, Xindong X, Jianhua F (2021). The specific connexin 43-inhibiting peptide Gap26 improved alveolar development of neonatal rats with hyperoxia exposure. Front Pharmacol.

[59] Elremaly W, Mohamed I, Rouleau T, Lavoie JC (2015). Adding glutathione to parenteral nutrition prevents alveolar loss in newborn guinea pig. Free Radic Biol Med.

[60] Shrestha AK, Gopal VYN, Menon RT, Hagan JL, Huang S, Shivanna B (2018). Lung omics signatures in a bronchopulmonary dysplasia and pulmonary hypertension-like murine model. Am J Physiol Lung Cell Mol Physiol.

[61] Sampath V, Garland JS, Helbling D (2015). Antioxidant response genes sequence variants and BPD susceptibility in VLBW infants. Pediatr Res.

[62] Delaney C, Wright RH, Tang J-R (2015). Lack of EC-SOD worsens alveolar and vascular development in a neonatal mouse model of bleomycin-induced bronchopulmonary dysplasia and pulmonary hypertension. Pediatr Res.

[63] Mathew R (2020). Signaling pathways involved in the development of bronchopulmonary dysplasia and pulmonary hypertension. Children (Basel).

[64] Speer CP (2006). Pulmonary inflammation and bronchopulmonary dysplasia. J Perinatol.

[65] Kuang PP, Zhang XH, Rich CB, Foster JA, Subramanian M, Goldstein RH (2007). Activation of elastin transcription by transforming growth factor-beta in human lung fibroblasts. Am J Physiol Lung Cell Mol Physiol.

[66] Nakanishi H, Sugiura T, Streisand JB, Lonning SM, Jesse D, Roberts J (2007). TGF-β-neutralizing antibodies improve pulmonary alveologenesis and vasculogenesis in the injured newborn lung. Am J Physiol-Lung Cell Mol Physiol.

[67] Ahlfeld SK, Wang J, Gao Y, Snider P, Conway SJ (2016). Initial suppression of transforming growth factor-β signaling and loss of TGFBI causes early alveolar structural defects resulting in bronchopulmonary dysplasia. Am J Pathol.

[68] Deng S, Zhang H, Han W, Guo C, Deng C (2019). Transforming growth factor-β-neutralizing antibodies improve alveolarization in the oxygen-exposed newborn mouse lung. J Interferon Cytokine Res.

[69] Luan Y, Zhang L, Chao S et al (2016) Mesenchymal stem cells in combination with erythropoietin repair hyperoxia-induced alveoli dysplasia injury in neonatal mice via inhibition of TGF-β1 signaling. Oncotarget 7(30):47082–47094 10.18632/oncotarget.9314 PMC5216925 27191651

[70] Sureshbabu A, Syed MA, Boddupalli CS (2015). Conditional overexpression of TGFbeta1 promotes pulmonary inflammation, apoptosis and mortality via TGFbetaR2 in the developing mouse lung. Respir Res.

[71] Thébaud B, Goss KN, Laughon M (2019). Bronchopulmonary dysplasia. Nat Rev Dis Primers.

[72] Carver BJ, Plosa EJ, Stinnett AM, Blackwell TS, Prince LS (2013). Interactions between NF-κB and SP3 connect inflammatory signaling with reduced FGF-10 expression. J Biol Chem.

[73] Thébaud B, Abman SH (2007). Bronchopulmonary dysplasia: where have all the vessels gone? Roles of angiogenic growth factors in chronic lung disease. Am J Respir Crit Care Med.

[74] Speer CP (2006). Inflammation and bronchopulmonary dysplasia: a continuing story. Semin Fetal Neonatal Med.

[75] Kunig AM, Balasubramaniam V, Markham NE (2005). Recombinant human VEGF treatment enhances alveolarization after hyperoxic lung injury in neonatal rats. Am J Physiol Lung Cell Mol Physiol.

[76] D’Angio CT, Maniscalco WM (2002). The role of vascular growth factors in hyperoxia-induced injury to the developing lung. Front Biosci.

[77] Le Cras TD, Markham NE, Tuder RM, Voelkel NF, Abman SH (2002). Treatment of newborn rats with a VEGF receptor inhibitor causes pulmonary hypertension and abnormal lung structure. Am J Physiol-Lung CelL Mol Physiol.

[78] Thébaud B, Ladha F, Michelakis ED (2005). Vascular endothelial growth factor gene therapy increases survival, promotes lung angiogenesis, and prevents alveolar damage in hyperoxia-induced lung injury: evidence that angiogenesis participates in alveolarization. Circulation..

[79] Syed MA, Choo-Wing R, Homer RJ, Bhandari V (2016). Role of nitric oxide isoforms in vascular and alveolar development and lung injury in vascular endothelial growth factor overexpressing neonatal mice lungs. PLoS One.

[80] Mokres LM, Parai K, Hilgendorff A (2010). Prolonged mechanical ventilation with air induces apoptosis and causes failure of alveolar septation and angiogenesis in lungs of newborn mice. Am J Physiol Lung Cell Mol Physiol.

[81] Kunzmann S, Seher A, Kramer BW (2008). Connective tissue growth factor does not affect transforming growth factor-beta 1-induced Smad3 phosphorylation and T lymphocyte proliferation inhibition. Int Arch Allergy Immunol.

[82] Kunzmann S, Speer CP, Jobe AH, Kramer BW (2007). Antenatal inflammation induced TGF-beta1 but suppressed CTGF in preterm lungs. Am J Physiol Lung Cell Mol Physiol.

[83] Wang X, Cui H, Wu S (2019). CTGF: a potential therapeutic target for Bronchopulmonary dysplasia. Eur J Pharmacol.

[84] Alapati D, Rong M, Chen S (2011). Connective tissue growth factor antibody therapy attenuates hyperoxia-induced lung injury in neonatal rats. Am J Respir Cell Mol Biol.

[85] Chen S, Rong M, Platteau A (2011). CTGF disrupts alveolarization and induces pulmonary hypertension in neonatal mice: implication in the pathogenesis of severe bronchopulmonary dysplasia. Am J Physiol Lung Cell Mol Physiol.

[86] Lal CV, Bhandari V, Ambalavanan N (2018). Genomics, microbiomics, proteomics, and metabolomics in bronchopulmonary dysplasia. Semin Perinatol.

[87] Syed M, Das P, Pawar A (2017). Hyperoxia causes miR-34a-mediated injury via angiopoietin-1 in neonatal lungs. Nat Commun.

[88] Ruiz-Camp J, Quantius J, Lignelli E et al (2019) Targeting miR-34a/Pdgfra interactions partially corrects alveologenesis in experimental bronchopulmonary dysplasia. EMBO Mol Med 11(3):e9448 10.15252/emmm.201809448 PMC6404112 30770339

[89] Das P, Syed MA, Shah D, Bhandari V (2018). miR34a: a master regulator in the pathogenesis of bronchopulmonary dysplasia. Cell Stress.

[90] Shah D, Das P, Alam MA (2019). MicroRNA-34a promotes endothelial dysfunction and mitochondrial-mediated apoptosis in murine models of acute lung injury. Am J Respir Cell Mol Biol.

[91] Gilfillan M, Das P, Shah D, Alam MA, Bhandari V (2020). Inhibition of microRNA-451 is associated with increased expression of macrophage migration inhibitory factor and mitgation of the cardio-pulmonary phenotype in a murine model of bronchopulmonary dysplasia. Respir Res.

[92] Robbins ME, Dakhlallah D, Marsh CB, Rogers LK, Tipple TE (2016). Of mice and men: correlations between microRNA-17 approximately 92 cluster expression and promoter methylation in severe bronchopulmonary dysplasia. Am J Physiol Lung Cell Mol Physiol.

[93] Durrani-Kolarik S, Pool CA, Gray A (2017). miR-29b supplementation decreases expression of matrix proteins and improves alveolarization in mice exposed to maternal inflammation and neonatal hyperoxia. Am J Physiol Lung Cell Mol Physiol.

[94] Lal CV, Olave N, Travers C et al (2018) Exosomal microRNA predicts and protects against severe bronchopulmonary dysplasia in extremely premature infants. JCI Insight 3(5):e93994 10.1172/jci.insight.93994 PMC5922295 29515035

[95] Alam MA, Betal SGN, Aghai ZH, Bhandari V (2019). Hyperoxia causes miR199a-5p-mediated injury in the developing lung. Pediatr Res.

[96] Olave N, Lal CV, Halloran B (2016). Regulation of alveolar septation by microRNA-489. Am J Physiol Lung Cell Mol Physiol.

[97] Rogers LK, Robbins M, Dakhlallah D (2015). Attenuation of miR-17∼92 cluster in bronchopulmonary dysplasia. Ann Am Thorac Soc.

[98] Velten M, Britt RD, Heyob KM (2012). Prenatal inflammation exacerbates hyperoxia-induced functional and structural changes in adult mice. Am J Physiol Regul Integr Comp Physiol.

[99] Chao C-M, Carraro G, Rako ZA (2020). Failure to down-regulate miR-154 expression in early postnatal mouse lung epithelium suppresses alveologenesis, with changes in Tgf-β signaling similar to those induced by exposure to hyperoxia. Cells..

[100] Mecham RP (2018). Elastin in lung development and disease pathogenesis. Matrix Biol.

[101] Danan C, Jarreau PH, Franco ML (2002). Gelatinase activities in the airways of premature infants and development of bronchopulmonary dysplasia. Am J Physiol Lung Cell Mol Physiol.

[102] Hilgendorff A, Parai K, Ertsey R (2011). Inhibiting lung elastase activity enables lung growth in mechanically ventilated newborn mice. Am J Respir Crit Care Med.

[103] Alvira CM, Morty RE (2017). Can we understand the pathobiology of bronchopulmonary dysplasia?. J Pediatr.

[104] Luo Y, Li N, Chen H (2018). Spatial and temporal changes in extracellular elastin and laminin distribution during lung alveolar development. Sci Rep.

[105] Mižíková I, Ruiz-Camp J, Steenbock H (2015). Collagen and elastin cross-linking is altered during aberrant late lung development associated with hyperoxia. Am J Physiol Lung Cell Mol Physiol.

[106] Thibeault DW, Mabry SM, Ekekezie II, Zhang X, Truog WE (2003). Collagen scaffolding during development and its deformation with chronic lung disease. Pediatrics..

[107] Mižíková I, Morty RE (2015). The extracellular matrix in bronchopulmonary dysplasia: target and source. Front Med.

[108] Mižíková I, Pfeffer T, Nardiello C (2018). Targeting transglutaminase 2 partially restores extracellular matrix structure but not alveolar architecture in experimental bronchopulmonary dysplasia. FEBS J.

[109] Kho AT, Bhattacharya S, Tantisira KG (2010). Transcriptomic analysis of human lung development. Am J Respir Crit Care Med.

[110] Perkel JM (2017). Single-cell sequencing made simple. Nature..

[111] Guo M, Du Y, Gokey JJ (2019). Single cell RNA analysis identifies cellular heterogeneity and adaptive responses of the lung at birth. Nat Commun.

[112] Ardini-Poleske ME, Clark RF, Ansong C (2017). LungMAP: the molecular atlas of lung development program. Am J Physiol-Lung CelL Mol Physiol.

[113] Ding J, Ahangari F, Espinoza CR (2019). Integrating multiomics longitudinal data to reconstruct networks underlying lung development. Am J Physiol Lung Cell Mol Physiol.

